# Biological sex differences in renin angiotensin system enzymes ACE and ACE2 regulate normal tissue response to radiation injury

**DOI:** 10.3389/fphys.2023.1191237

**Published:** 2023-05-19

**Authors:** Guru Prasad Sharma, Anne Frei, Brian Fish, Tracy Gasperetti, Dana Veley, Nathan Szalewski, Austen Nissen, Heather A. Himburg

**Affiliations:** ^1^ Department of Radiation Oncology, Medical College of Wisconsin, Milwaukee, WI, United States; ^2^ Cancer Center, Medical College of Wisconsin, Milwaukee, WI, United States

**Keywords:** ACE, ACE2, biological sex, radiation pneumonitis, renin angiotensin system, medical countermeasures, myeloid cells, GI-ARS

## Abstract

**Introduction:** In experimental animal models, biological sex-differences in the manifestation and severity of normal tissue radiation injury have been well-documented. Previously we demonstrated male and female rats have differential and highly reproducible responses to high-dose partial body irradiation (PBI) with male rats having greater susceptibility to both gastrointestinal acute radiation syndrome (GI-ARS) and radiation pneumonitis than female rats.

**Methods:** In the current study, we have investigated whether differential expression of the renin-angiotensin system (RAS) enzymes angiotensin converting enzyme (ACE) and ACE2 contribute to the observed sex-related differences in radiation response.

**Results:** During the period of symptomatic pneumonitis, the relative ratio of ACE to ACE2 (ACE/ACE2) protein in the whole lung was significantly increased by radiation in male rats alone. Systemic treatment with small molecule ACE2 agonist diminazene aceturate (DIZE) increased lung ACE2 activity and reduced morbidity during radiation pneumonitis in both sexes. Notably DIZE treatment also abrogated morbidity in male rats during GI-ARS. We then evaluated the contribution of the irradiated bone marrow (BM) compartment on lung immune cell infiltration and ACE imbalance during pneumonitis. Transplantation of bone marrow from irradiated donors increased both ACE-expressing myeloid cell infiltration and immune ACE activity in the lung during pneumonitis compared to non-irradiated donors.

**Discussion:** Together, these data demonstrate radiation induces a sex-dependent imbalance in the renin-angiotensin system enzymes ACE and ACE2. Additionally, these data suggest a role for ACE-expressing myeloid cells in the pathogenesis of radiation pneumonitis. Finally, the observed sex-differences underscore the need for consideration of sex as a biological variable in the development of medical countermeasures for radiation exposure.

## Introduction

Since the 11 September 2001 terrorist attacks, the federal government has recognized the possibility of attacks involving radiological or nuclear weapons as a national security priority.

Victims of radiation exposure may experience a range of dose dependent toxicities which include both the acute syndromes of radiation sickness as well as delayed effects of radiation exposure (DEARE). Our laboratory has developed a rat partial body irradiation (PBI) model which recapitulates the major sequelae of acute (gastrointestinal and hematopoietic) and delayed (lung and kidney) radiation injuries (Fish et al., 2021). In this model, we previously reported sex-differences in morbidity during the sequelae of gastrointestinal acute radiation syndrome (GI-ARS) and lung-DEARE following PBI doses of 10–14 Gy with adult male rats having increased susceptibility to both GI-ARS and lung-DEARE than age-matched female rats (Fish et al., 2021). However, the underlying biological mechanisms regulating these observed sex-related differences in radiation sensitivity are unknown.

The observed sex difference in lung-DEARE in our rodent model mirrors the established sex disparity in COVID-19 respiratory syndrome patients where globally men have significantly higher death rates than women (Gagliardi et al., 2020; Mukherjee and Pahan, 2021). Interestingly, there are other established commonalities between radiation pneumonitis and COVID-19 acute respiratory syndrome including immune infiltration, inflammatory cytokine release, and dysregulation of the renin-angiotensin system (RAS) (Barhoumi et al., 2021; Rios et al., 2021; Geng et al., 2022). It has been hypothesized that the COVID-19 sex disparity may be attributable to dysregulation of RAS as the angiotensin converting enzyme (ACE) family member ACE2 serves as the entry point for the SARS-CoV-2 virus (Miličić Stanić et al., 2021). SARS infection both inhibits ACE2 activity and decreases ACE2 expression in infected cells (Imai et al., 2005; Haga et al., 2008). Since ACE2 directly counteracts ACE by converting vasoconstrictor angiotensin II (AngII) to angiotensin (1–7), the loss of ACE2 has been proposed to drive an imbalance in favor of excess AngII levels (Sriram and Insel, 2020). In radiation injury, several decades of research support the usage of angiotensin converting enzyme (ACE) inhibitors for mitigation of a range of radiation-induced toxicities including: acute hematopoietic injury (McCart et al., 2019), pneumonitis (Medhora et al., 2012; Gasperetti et al., 2021; Mungunsukh et al., 2021), cardiac fibrosis (van der Veen et al., 2015), optic neuropathy (Kim et al., 2004; Ryu et al., 2007), and nephropathy (Moulder et al., 2007; Fish et al., 2016). Recently, we have also demonstrated pharmacologic ACE2 agonism with the small molecule diminazene aceturate (DIZE) promotes survival in rodent models of hematopoietic acute radiation syndrome (H-ARS) and DEARE (Gasperetti et al., 2022).

ACE inhibitors such as lisinopril have well-established function as vasodilators and suppressors of reactive oxygen species production in vascular pharmacology (Schramm et al., 2012). While these angio-protective effects are likely to promote multi-organ recovery following radiation injury, we have previously demonstrated ACE inhibitor treatment also directly regulates the function of irradiated immune cells (Sharma et al., 2022). Treatment with ACE inhibitor lisinopril suppressed the infiltration of inflammatory immune cell populations in the lung and the secretion of pro-inflammatory cytokines MCP-1 (CCL2) and MIP1a (CCL3) (Sharma et al., 2022). Importantly, radiation injury induced an increase in the expression and activity of ACE in subsets of lung infiltrating CD45^+^CD11b^+^ myeloid cells that was abrogated by treatment with lisinopril (Sharma et al., 2022).

In the current study, we have assessed whether an imbalance in the RAS within the immune compartment of male rats relative to female rats may play a role in the observed sex-difference in radiation sensitivity. Here, we report sex differences in the regulation of the RAS enzymes ACE and ACE2 in response to radiation in both the lung and bone marrow. We demonstrate the agonism of ACE2 mitigates lung injury in both sexes. Additionally, we demonstrate using a bone marrow transplantation model that concurrent injury to the bone marrow exacerbates recruitment of ACE-expressing myeloid cells to the lung during pneumonitis.

## Materials and methods

### Experimental animals

All studies described were performed in accordance with an approved Institutional Animal Care and Use Committee protocol. Age and sex-matched WAG/RijCmcr rats were bred and maintained in a barrier facility at our institution. Two weeks prior to irradiation, rats were switched to a moderate antioxidant diet (Teklad Global 2018 diet) which is more representative of antioxidant levels in a human diet (Gasperetti et al., 2021). All rats were provided reverse osmosis hyper-chlorinated water *ad libitum*.

### Partial body irradiation (PBI) model

Adult female and male WAG/RijCmcr rats were exposed to 12.5 partial body irradiation (PBI) with bone marrow shielding to one hind-limb (X-RAD 320 Precision, 320 kVp; 169 cGy/min). All rats received supportive care post radiation consisting of antibiotics (enrofloxacin ∼10 mg/kg/day) in the drinking water from days 2–14, subcutaneous saline (40 ml/kg) days 2–10, and powdered diet days 35–70. All irradiations were performed between 7–10 am.

### Diminazene aceturate (DIZE) administration

Diminazene aceturate (DIZE) was purchased from Sigma-Aldrich (St. Louis, Missouri, catalog #D7770) and MedChemExpress (catalog #HY-12404, Monmouth Junction, NJ) and reconstituted in sterile water at a stock concentration of 10 mg/ml. Rats were weighed immediately prior to drug administration and administered 15 mg/kg of the 10 mg/ml DIZE solution via subcutaneous injection (Qi et al., 2013). Vehicle control rats received subcutaneous injections of an equivalent volume of sterile water. Rats were dosed subcutaneously starting at 72 h post-PBI with either 15 mg/kg DIZE or sterile water (3X/week (MWF) for the duration of the study.

### Bone marrow transplantation model

Three donor adult female WAG/RijCmcr rats (11–12 weeks old) were exposed to 7.75 Gy total body irradiation (TBI) or sham irradiation. On day 30 post-irradiation, donor animals were humanely euthanized and both femurs were carefully excised. Bone marrow from individual femurs was flushed using a 28-gauge syringe into 5 ml of sterile phosphate buffered saline (PBS) containing 10% fetal bovine serum. Cells were pelleted by centrifugation and the red blood cell fraction was removed with (Ammonium Chloride Potassium) ACK lysis buffer. Total viable cells were determined using a Countess Automated Cell Counter Hemocytometer C10227 (Invitrogen) with trypan blue exclusion method. Adult female WAG/RijCmcr rats bone marrow recipients were conditioned with 13 Gy TBI. All rats were irradiated without the use of anesthetics by being placed in a plastic jig and the entire body was exposed using a XRAD 320 kV orthovoltage x-ray system (PrecisionX-Ray, Madison, CT). The X-ray system was operated at 320 kVp and13 mAs with a half-value layer of 1.4 mm copper and a dose rate of 169 cGy/min. Recipient rats received 5 × 10^6^ whole bone marrow cells in a volume of 300 ul via intravenous tail vein injection at 24 h post-irradiation.

### Peripheral blood analysis

Complete blood counts complete blood counts (CBC) as done previously (Gasperetti et al., 2022). Briefly, rats were restrained with one hand by positioning the forelimbs in the caudodorsal direction with the thumb and middle finger. A 23-gauge needle attached to a syringe was carefully inserted into either the right or left external jugular vein and blood was collected. Syringes were coated with EDTA to prevent clotting for all CBC blood draws. Complete blood counts were determined using a Heska Element 5 Veterinary Hematology Analyzer. The remaining blood was centrifuged to separate plasma for further analysis**.** Plasma samples were shipped to Eve Technologies for progesterone and testosterone measurement using the Steroid/Thyroid 6-Plex Discovery Assay (Eve Technologies). Plasma samples were analyzed ELISA for Ang (1–7) levels and Ang II at day 70 following 12.5 Gy PBI injury. The commercial kits: Ang II ELISA kit: (Cat# MBS730655, MyBioSource) and Ang (1–7) ELISA kit: (Cat# MBS8806649, MyBioSource) were used and followed the protocol per the manufacturer instructions.

### Breathing rate measurement

Breathing rates were measured using a MouseOx Plus Pulse Oximeter (Starr Life Sciences Corp) in accordance with manufacturer’s instructions. Rats were acclimated to the recording processing on the day prior to recording. After calibration for movement, the breathing rate was measured over a five-minute period and averaged to obtain the reported rate for each rat.

### Bone marrow (BM) isolation

Bone marrow was harvested from the femurs of euthanized rats. Each femur was carefully excised from the animal and flushed with PBS containing 10% FBS and 1% Pen/Strep using a 1 ml syringe with a 25G needle, making sure to flush from both ends to ensure all cells were removed. Cells were pelleted by centrifugation and the red blood cell fraction was removed with ACK lysis buffer. Total viable cells were determined using a Countess Automated Cell Counter Hemocytometer C10227 (Invitrogen) with trypan blue exclusion method.

### Lung tissue dissociation

Lung cells were dissociated as previously described (Sharma et al., 2022). The right lung was dissociated to a single cell suspension with Multi Tissue Dissociation Kit 2 (130–110–203, Miltenyi) according to the manufacturer’s protocol using the gentle MACS Dissociator (Miltenyi). The cell suspension was filtered through a 70 μm strainer, pelleted by centrifugation, and cleared of red blood cells with ACK Red Blood Cell Lysis buffer (BP10-548E, Lonza). Live cells were enumerated using trypan blue exclusion counting on a Countess Automated Cell Counter Hemocytometer C10227 (Invitrogen). Lung cells were then used as described below for Fluorescence-Activated Cell Sorting (FACS) analysis. Additionally, ten million lung cells per animal were used for CD45^+^ enrichment using the CD45 MicroBeads kit (cat# 130–109–682, Miltenyi) per manufacturer’s instructions.

### Flow cytometry analysis

Flow cytometry analysis was performed using one million cells. 7-AAD (420404, Bio Legend) was used to exclude dead cells. Single color tubes were used to set up a compensation matrix and a Fluorescence Minus One (FMO) control was included to ensure specific staining. Antibody staining was performed at 4°C for 30 min in staining buffer (1XPBS with 2% FBS). The following anti-rat antibodies were used: CD45-APC (17–0461–82, Thermo Fisher), CD11b- PE/CY7 (201818, Bio Legend), PE-CD45RA (130–106–774, Miltenyi), and CD3-PE (12–0030-82). Sample data were acquired on a MACSQuant 10 Analyzer Flow Cytometer (Miltenyi) and analyzed using FlowJo software version 10.0 (BD Life Sciences). To verify gating and purity, all populations were routinely backgated.

### ACE and ACE2 activity assay

Assays were performed as published before (Gasperetti et al., 2022) using commercially available fluorometric ACE (Sigma-Aldrich, CS0002) and ACE2 (Sigma-Aldrich, MAK377) kits as per manufacturer protocol. Two million cells were lysed in 0.2 mL lysis buffer supplied within the kit and protein concentration was determined using the BCA method. The data are represented as units of activity per microgram of protein where one unit (U) is the amount of enzyme that catalyzes the reaction of 1 nmol of substrate per minute under standard conditions.

### RT-PCR analysis

RNA isolation was performed using RNeasy Micro Kit (cat#74004, Qiagen). RNA was quantified using a Nanodrop 2000 Spectrophotometer (model- Model: 840–274200, Thermo Fisher Scientific). The synthesis of cDNA was done using the High-Capacity RNA-to-cDNA™ Kit (cat# 4387406, Thermo Fisher Scientific). Transcript expression was analyzed in triplicate using TaqMan Gene Expression Assay primer probes (cat#4331182, Thermo Fisher Scientific). The primer probes used were *Ace* (Rn00561094_m1), *Ace*2 (Rn01416293_m1) and the reference *Gapdh* (Rn01775763_m1). Expression of target genes was normalized to GAPDH. Expression of target genes was normalized to GAPDH. The relative expression of the gene was calculated with respect to control (0 Gy) and presented as fold change (2^-(ΔCt subject)-(mean ΔCt control)), statistics done on ΔCt values.

### Western blotting

Tissue lysis and protein analysis by western blotting were performed using established methodologies (Sharma et al., 2019; Gasperetti et al., 2021). Briefly, lung and kidney tissues (∼50 mg) were lysed using Qiagen tissue lysate method in RIPA buffer (20–188, Millipore Sigma) containing protease and phosphatase inhibitors. Total protein was estimated using BCA method (23225, Pierce™). Twenty μg protein from each homogenized sample was boiled for 5 min in Laemmli sample buffer (Bio-Rad), separated on a precasted gradient polyacrylamide gel (NP0335BOX, NuPAGE) electrophoreses (PAGE) (Bio-Rad), and transferred to a PVDF membrane (IPVH00010, Millipore). The blots were blocked for 1 h at room temperature in 5% fat free milk and probed with ACE (ab254222, Abcam), ACE2 (MA5-35544, Thermo Fisher Scientific) and GAPDH (ab181602, Abcam) primary antibodies at 4°C overnight. On the following day, the membrane was washed and incubated with secondary antibody conjugated to horseradish peroxidase (31460, Thermo Fisher Scientific). The membrane was washed with 0.5% PBST and signal was observed using Pico substrate (32132, Pierce) on a Bio-Rad chemiluminescence machine. Densitometric band intensity was determined using Image J analysis software. Representative cropped images are presented in the main figure. Uncropped blots are provided in the Supplementary Data.

### Histology

Histological analysis was performed on femur and lungs on day 70 following 12.5 Gy PBI. Tissue processing and staining were performed at the MCW Children’s Research Institute (CRI) Histopathology Core. The left lung inflated and fixed in 10% paraformaldehyde prior to paraffin embedding. Lungs were then sectioned (4 μm thick) and stained with tryptase antibody (IMGENEX catalogue #IMG-80250, 1:150) or Masson’s trichrome stain (Fish et al., 2021). Femurs were decalcified, sectioned (4 μm thick) and stained with hematoxylin and eosin (H&E). Mast cells were assessed by positive tryptase staining in five randomly selected fields (10x) using ImageJ software. Collagen quantification was performed on five 20X fields per tumor in ImageJ using the ImageJ “Colour Deconvolution” tool with the “Masson’s trichrome” setting.

### Graphic design

Schematics were “Created with BioRender.com” using an academic license.

### Statistical analysis

Statistical analysis was performed using the GraphPad Prism version 9 (GraphPad Software, Inc.). Data are represented as mean +/- standard error (SEM). Statistical analysis of multiple groups and time points was conducted using ANOVA with appropriate multiple comparison tests. *p* values < 0.05 were considered statistically significant.

## Results

### Characterization of peripheral blood ACE activity following partial body irradiation (PBI)

**FIGURE 1:**
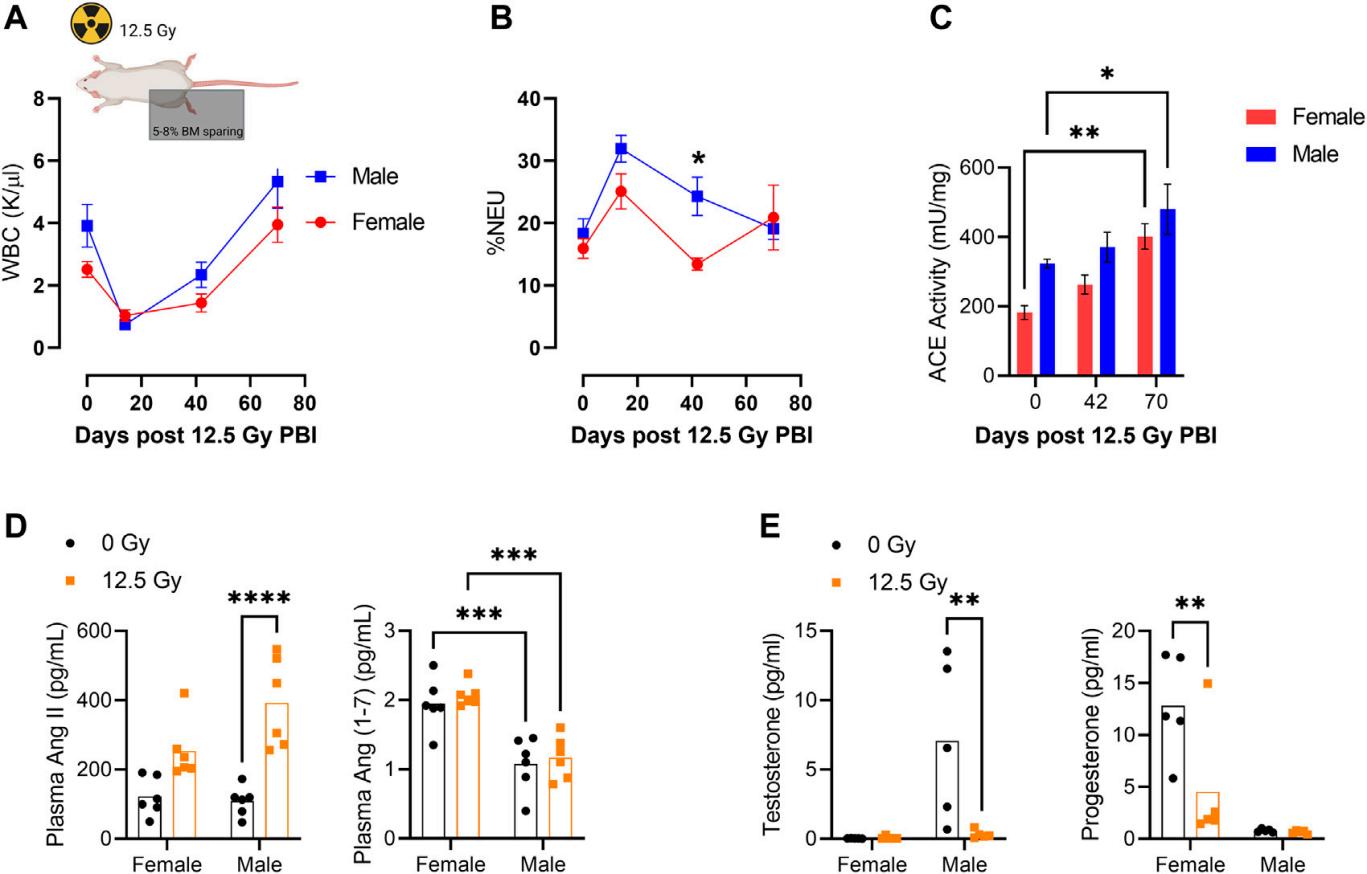
Characterization of Peripheral Blood ACE Activity Following Partial Body Irradiation (PBI). **(A)** Total peripheral blood white blood cell (WBC) counts and **(B)** percentage peripheral blood neutrophils (%NEU) at days 0, 14, 42, and 70 following 12.5 Gy partial body irradiation (PBI) with shielding of 5%–8% BM in one hind limb. **(C)** ACE activity within CD45^+^ peripheral blood mononuclear cells (PBMCs) at days 0, 42, and 70 following 12.5 Gy PBI. **(D)** Plasma AngII and Ang(1-7) levels at day 70 following irradiation. (N = 5 rats per group). **(E)** Blood plasma levels of testosterone and progesterone at day 70 following 12.5 Gy. (N= 5 rats per group). For all graphs, error bars indicate standard error of the mean *p < 0.05; **p < 0.01.

Male and female rats were exposed to a dose of 12.5 Gy PBI to allow for tissue collection at scheduled time points coinciding with acute hematologic injury (D14), immediately prior to symptomatic pneumonitis (D42) and during symptomatic pneumonitis (D70). Total peripheral white blood cell (WBC) counts were decreased following radiation injury and required up to 42 days to recover to pre-irradiation levels (Figure 1A). Interestingly, at day 42 irradiated male rats exhibited an elevation in the percentage of circulating neutrophils relative to female rats (Figure 1B). Within the peripheral blood CD45^+^ cell population, we then assessed ACE enzymatic activity. ACE activity increased in the peripheral blood CD45^+^ population after irradiation in both male and female rats and was significantly different in both sexes compared to baseline at day 70 (Figure 1C). Day 70 plasma was also analyzed for circulating levels of RAS peptides AngII and Ang (1–7) (Figure 1D). Plasma AngII levels were similar between males and females, but were increased post-irradiation in both male and female rats (Figure 1D). Conversely, plasma Ang (1–7) levels were unchanged by radiation but significantly higher in females than males at both timepoints (Figure 1D). It is notable that this radiation dose renders both male and female rats sterile and circulating levels of testosterone and progesterone were respectively absent by day 70 post injury (Figure 1E).

### Sex differences in lung immune infiltrate during radiation pneumonitis

**FIGURE 2:**
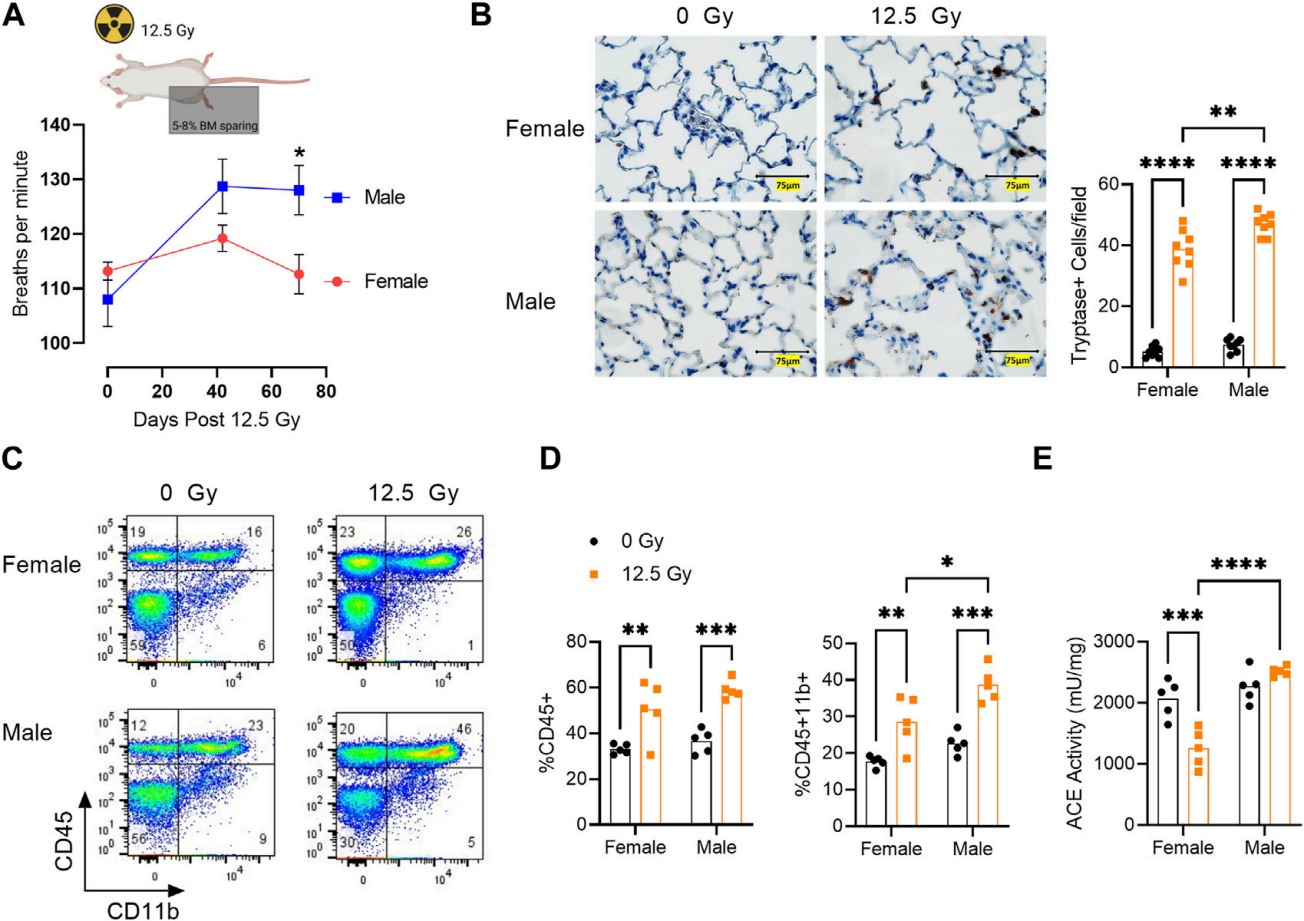
Sex Differences in Lung Immune Infiltrate During Radiation Pneumonitis. **(A)** Breathing rate at days 42 and 70 following 12.5 Gy Partial Body Irradiation (PBI). **(B)** Left, representative lung sections stained with mast cell marker tryptase (brown) in 0 Gy and 12.5 Gy PBI male and female rats at 70 days post radiation. Right, quantification of tryptase^+^ mast cells. **(C)** Representative flow cytometric analysis of the lung CD45^+^CD11b^+^ myeloid cell population at day 42 post-irradiation. **(D)** Quantification of percentage lung CD45^+^ cells and percentage CD45^+^ CD11b^+^ myeloid cells. **(E)** ACE activity within the lung CD45^+^ cell population at day 42 following 12.5Gy PBI. (N = 5 rats/group). For all graphs, error bars indicate standard error of the mean *p < 0.05; **p < 0.01; ***p < 0.001; ****p < 0.0001.

We then evaluated lung function and immune infiltration during pneumonitis. Following irradiation, breathing rate in male rats was elevated relative to females and was significantly increased at day 70 post-PBI (Figure 2A). Histological analysis of the lung was performed to quantify tryptase^+^ mast cells (Figure 2B). Mast cells are a subset of myeloid cells recruited to sites of inflammation (Krystel-Whittemore et al., 2015) and have previously been shown to be recruited to rat lung following high dose irradiation to the thorax (Ward et al., 1993; Szabo et al., 2010). Consistent with these reports, we observe a significant increase in tryptase^+^ mast cells in both sexes post-irradiation. The increase in tryptase^+^ mast cells was significantly greater in males than females (Figure 2B). Mast cell invasion in the lung is associated with collagen deposition and the subsequent development of radiation fibrosis in the rat lung (Watanabe et al., 1974; Ward et al., 1990). A small but significant increase in collagen deposition assessed by Masson’s trichrome staining was observed at day 70 following radiation in both sexes (Supplementary Figure S1).

Flow cytometric analysis demonstrated total CD45^+^ immune cells and CD45^+^CD11b^+^ myeloid cells were increased following irradiation in the lungs of both female and male rats (Figure 2C). Although the percentage of CD45^+^ cells post-irradiation was similar between the sexes, male rats exhibited an enrichment for CD45^+^CD11b^+^ myeloid cells compared to female rats (Figure 2D)**.** Interestingly, ACE activity within the lung CD45 population was differentially regulated by radiation in male and female rats. In females, ACE activity decreased from day 0 to day 42 post-irradiation, whereas ACE activity was not significantly altered in male rats at this time point. Post-irradiation, ACE activity in the lung CD45^+^ population was significantly greater in males *versus* females (Figure 2E).

### Whole lung ACE and ACE2 expression

**FIGURE 3:**
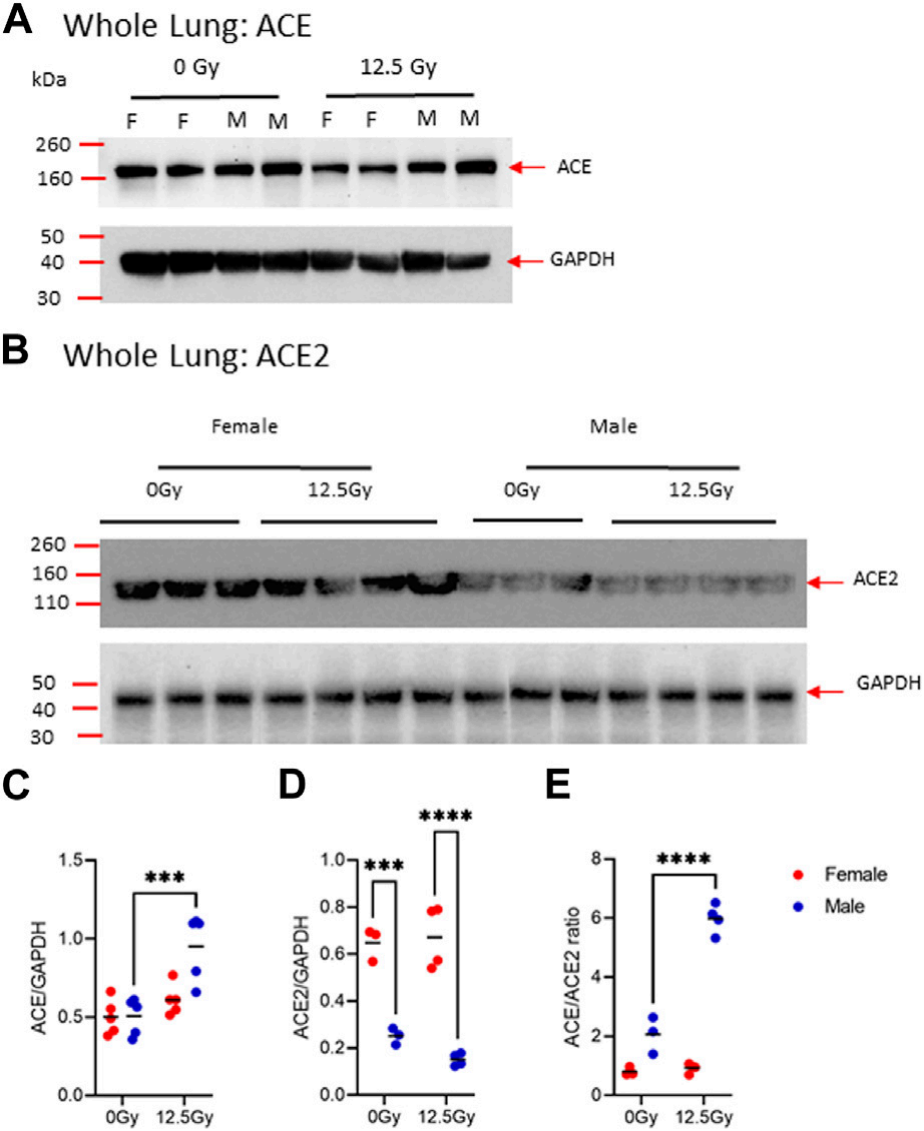
Radiation-induced changes in ACE and ACE2 protein expression in the whole lung. **(A)** Representative, cropped day 70 western blot analysis of ACE **(A)** and ACE2 **(B)** protein in whole lung lysates in 0 Gy and 12.5 Gy partial body irradiated rats with corresponding GAPDH control. Uncropped blots are provided in Supplementary Figure S3. Western blotting bands of ACE **(C)** and ACE2 **(D)** were quantified by densiometric analysis and normalized with respect to GAPDH bands. **(E)** The ratio of ACE and ACE2 protein expression. (N = 5 rats/group). For all graphs, error bars indicate standard error of the mean ***p < 0.001; ****p < 0.0001.

To evaluate whether radiation alters the balance of ACE enzymes ACE and ACE2, we next performed PCR and Western blot analysis on whole lung lysates for ACE and ACE2 on day 70. At the transcript level, neither *Ace* or *Ace2* was significantly different between male and female rats **(** Supplementary Figure S2 **)**. However, total ACE protein was significantly increased following radiation in male rats alone (Figures 3A–C, Supplementary Figure S3). Total ACE2 protein was elevated in female rats compared to male rats at both baseline and following radiation injury (Figures 3B–D, Supplementary Figure S3). Based on these data, we next calculated the ratio of ACE/ACE2 protein to assess for overall imbalances in the ACE/ACE2 levels. Here, we observed the ratio ACE/ACE2 protein ratio is significantly increased by radiation in males alone (Figure 3E). In male rats, the ACE/ACE2 ratio is elevated from a mean of 2.1 prior to radiation to 5.9 at day 70 post-PBI.

### ACE2 agonism with diminazene aceturate (DIZE) improves survival following partial body irradiation

**FIGURE 4:**
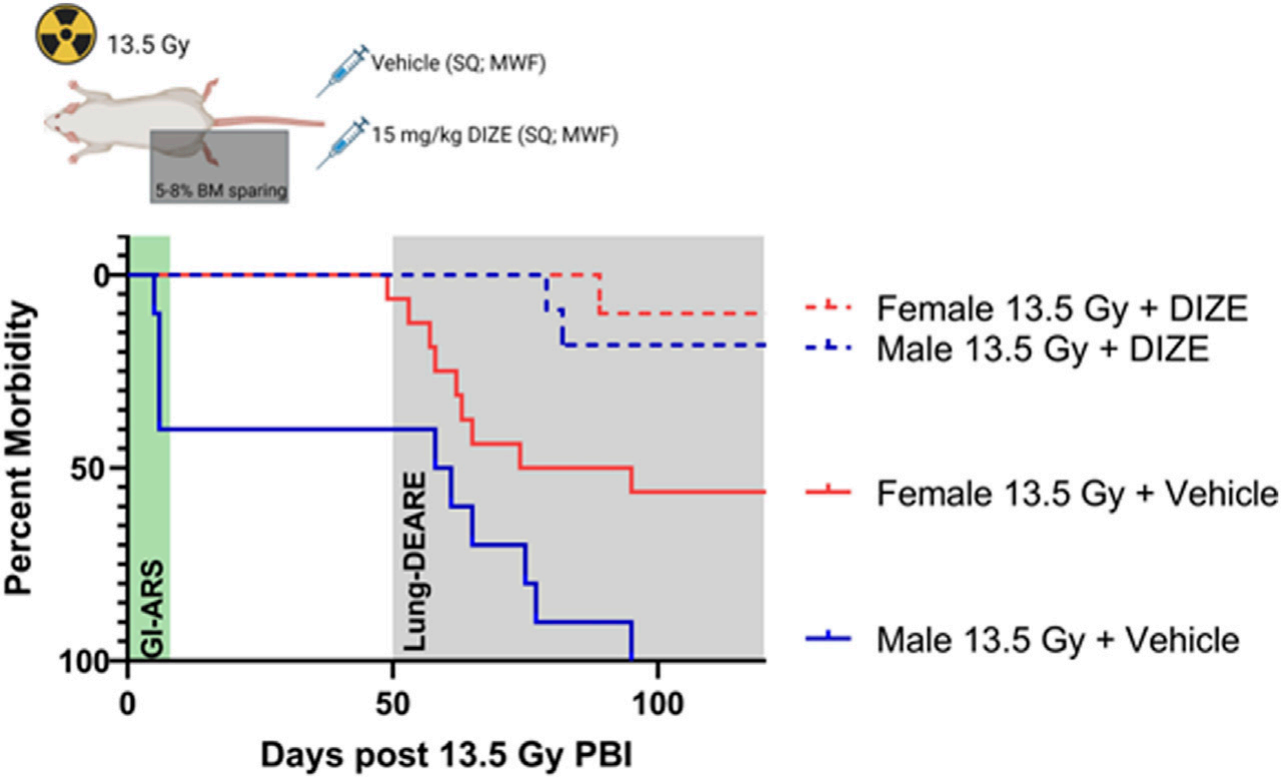
Treatment with ACE2 agonist diminazene aceturate (DIZE) reduces morbidity following 13.5 Gy partial body irradiation (PBI). Survival following 13.5 Gy PBI in male and female rats following subcutaneous (SQ) treatment with vehicle or DIZE three times per week (MWF) starting at 72 h post-irradiation and continued through endpoint. Log-rank analysis was used to compare survival between male vehicle (N = 10 rats), female vehicle (N = 16 rats), male + DIZE (N= 11 rats) and female + DIZE (N = 10 rats) through day 120.

In this rat strain, we have previously demonstrated sex-differences in susceptibility to two subsyndromes of radiation injury: gastrointestinal acute radiation syndrome (GI-ARS) and radiation pneumonitis (Gasperetti et al., 2022). Here, we evaluated if sex differences in radiation response could be abrogated by pharmacologically correcting for the imbalance in ACE/ACE2 ratio by treatment with the small molecule ACE2 agonist diminazene aceturate (DIZE). Male and female rats were exposed to 13.5 Gy PBI and treated subcutaneously with either vehicle or DIZE through endpoint (day 120). Increased whole lung ACE2 activity was confirmed in a satellite cohort of male rats treated with DIZE (Supplementary Figure S4)**.** Consistent with our prior studies, survival in vehicle-treated male rats was reduced compared to female rats due to a combination of increased GI-ARS morbidity (deaths prior to day 8) and increased in lung morbidity (deaths days 50–120) (Figure 4). Median survival was 59.5 days in vehicle-treated male rats *versus* 84.5 days in vehicle-treated female rats. Administration of DIZE improved survival in both groups (Figure 4). In DIZE-treated males no deaths were observed before day 8 (0/11 rats) compared to four deaths (4/10) in the male vehicle group. The remainder of the male vehicle rats succumbed to morbidities associated with respiratory failure during days 50–120. However, in the DIZE-treated group, 82% (9/11) rats survived past day 120. Log-rank analysis of the all-cause morbidity in DIZE-treated males compared to vehicle control was highly significant (*p* < 0.0001). In female rats, no morbidity was observed during GI-ARS, but 56% (9/16) of vehicle treated females succumbed to lung injury. Similar to males, DIZE treatment was capable of mitigating lung injury in females with 90% (9/10) surviving to day 120 (*p* = 0.017).

### Bone marrow ACE and ACE2 expression

**FIGURE 5:**
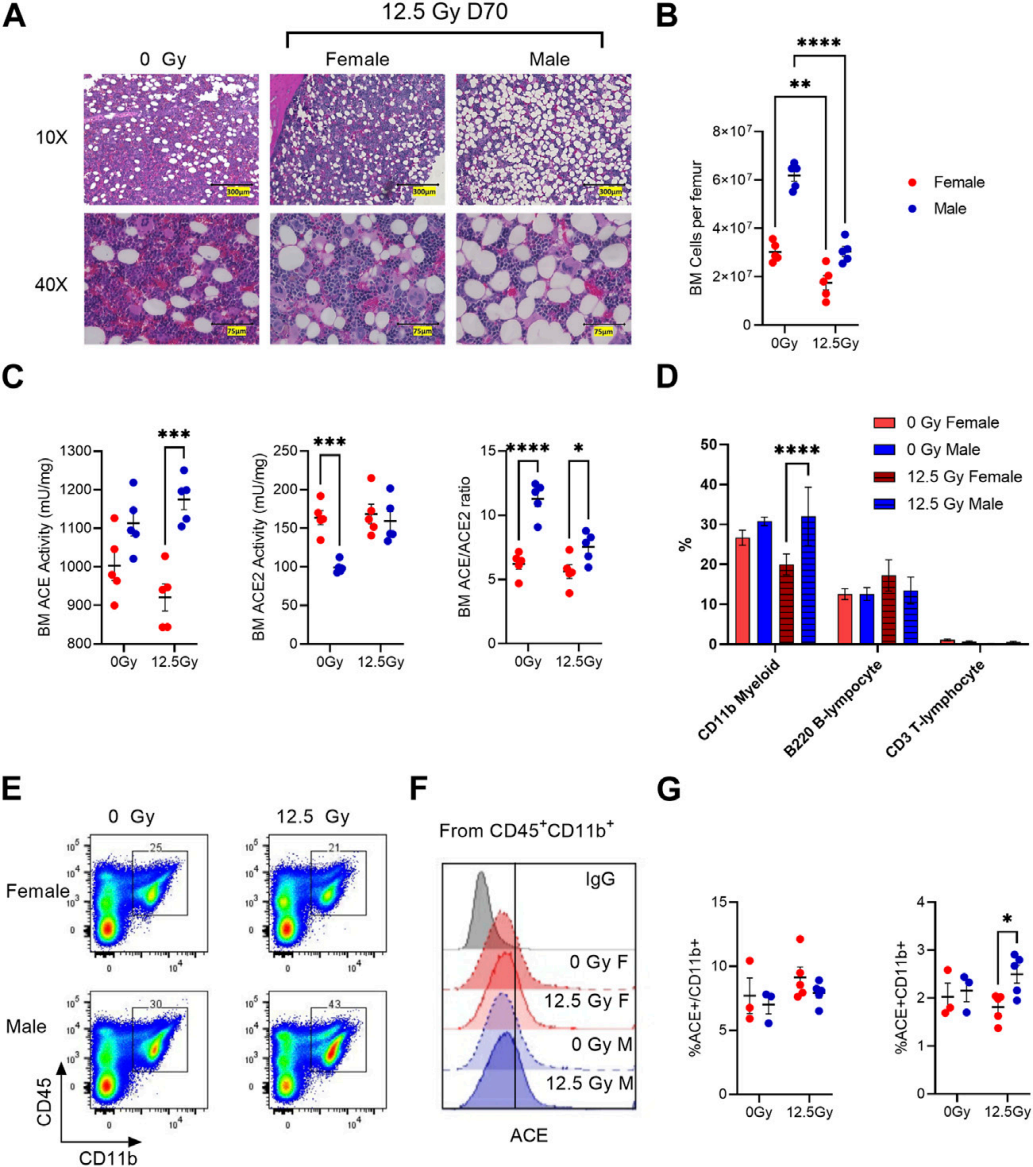
Radiation-induced changes in ACE and ACE2 protein expression in the bone marrow (BM). **(A)** Representative H&E-stained sections of male and female femurs prior to irradiation (0 Gy) and at day 70 following 12.5 Gy PBI. **(B)** Total viable bone marrow cells per femur at day 70 in non-irradiated control and irradiated rats. **(C)** BM enzymatic activity of ACE (left) and ACE2 (middle) at day 70. Right, calculated ratio of ACE/ACE2 at day 70. **(D)** Percent of total bone marrow positive for mature bone marrow lineage markers: CD11b, B220 and CD3 markers in control and irradiated rats at day 70. **(E)** Representative flow cytometry of BM CD45^+^CD11b^+^ myeloid cell population. **(F)** Representative histograms of ACE cell surface staining within the BM CD45^+^CD11b^+^ population. **(G)** Left, quantification of ACE^+^ expression within the CD45^+^CD11b^+^ population. Right, quantification of ACE^+^CD45^+^CD11b^+^ population. N = 5 rats/group. For all graphs, error bars indicate standard error of the mean *p < 0.05; **p < 0.01; ***p < 0.001; ****p < 0.0001.

Since our prior data indicate radiation increases ACE activity within myeloid cell fractions *ex vivo*, we next evaluated whether radiation induces long term imbalances in ACE and ACE2 activity within the bone marrow (BM). Here we observed both female and male rats exhibit a long-term loss in BM cellularity following irradiation (Figures 5A, B **)**. Within the whole BM, we assessed differences in total ACE and ACE2 activity following irradiation. Here we saw higher BM ACE activity in male rats compared to female rats both at baseline and at day 70 post-irradiation, with the difference reaching statistical significance at day 70 (Figure 5C). BM ACE2 activity was initially greater in female rats relative to male rats, but ACE2 activity in males and females was similar at day 70 post-irradiation (Figure 5C). The ratio of BM ACE/ACE2 activity is significantly higher in male rats both at baseline and day 70 post-PBI compared to female rats (Figure 5C). To see what lineage cell subsets are potentially contributing to increased ACE activity in male rats, we performed FACS analysis for specific BM lineages including CD11b^+^ myeloid cells, B220^+^ B-lymphocytes, and CD3^+^ T-lymphocytes. In irradiated male rats, the proportion of CD45^+^CD11b^+^ BM myeloid cells were increased relative to female rats at day 70 (Figure 5D). No significant differences in B or T lymphocytes were observed (Figure 5D). We then assessed ACE expression within the BM CD45^+^CD11b^+^ myeloid cell population (Figure 5E). 5%-10% of CD11b BM cells were positive for cell surface ACE by FACS analysis (Figure 5F). No differences in the fraction of CD11b cells expressing ACE were observed between sexes or following radiation (Figure 5F). However, the total fraction of ACE^+^CD11b cells was higher in irradiated males compared to irradiated females due to the overall increase in CD11b cells observed in irradiated males (Figure 5G).

### Transplantation of irradiated bone marrow increases lung ACE activity

**FIGURE 6:**
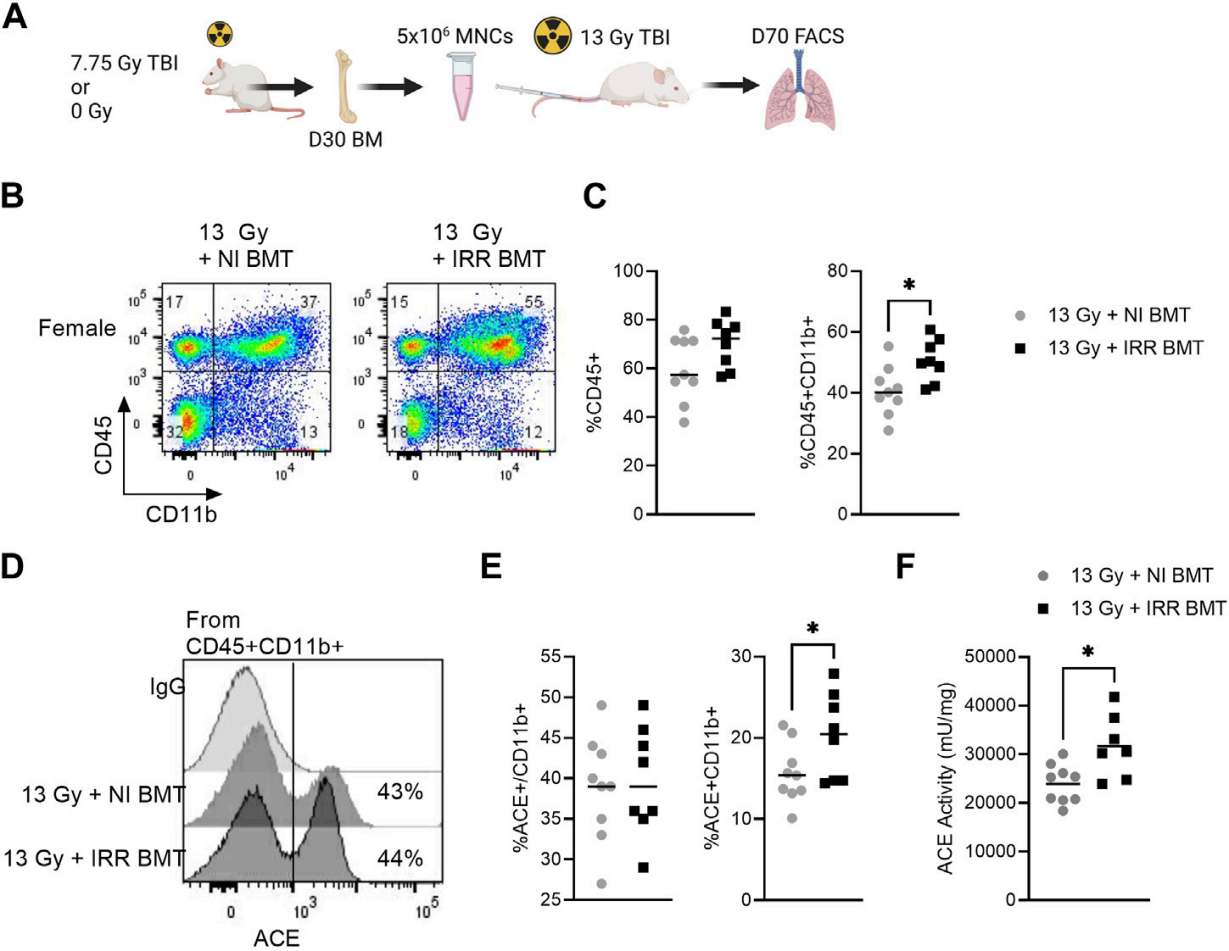
Recipients of irradiated bone marrow exhibit elevated myeloid ACE activity in the whole lung. **(A)** Bone marrow mononuclear cells (BM MNCs) from either control (0 Gy) or 7.75 Gy irradiated donors were transplanted into recipient rats exposed to 13 Gy. At day 70, the lung myeloid compartment was assessed by FACS analysis. **(B)** Representative flow cytometry gating of the CD45^+^CD11b^+^ myeloid cells in the lungs of BM-recipient rats. **(C)** Left, quantification of total lung CD45^+^ and CD45^+^ CD11b^+^ cells. **(D)** Representative histograms of ACE^+^ cells within the CD45^+^CD11b^+^ myeloid cell fraction. **(E)** Left, quantification of ACE^+^ expression within the CD45^+^CD11b^+^ population. Right, quantification of ACE^+^CD45^+^CD11b^+^ population. **(F)** Whole lung enzymatic ACE activity. (N = 9 rats/group). For all graphs, *p < 0.05.

In the partial body irradiation injury model, it is possible the observed long-term BM damage may lead to systemic immune dysfunction. To isolate the contribution of the irradiated BM on lung immune cell infiltration and ACE imbalance during pneumonitis, we performed a BM assay in female rats exposed to 13 Gy total body irradiation. Rats were transplanted with 5 × 10^6^ BM mononuclear cells (BM MNCs) from either healthy donors or from donors on day 30 following exposure to 7.75 Gy total body irradiation (Figure 6A). Recipient rats were euthanized on day 70 to assess for changes in lung immune infiltrate and ACE activity. Although equal BM cell doses were transplanted in each group, rats receiving irradiated BM MNCs had lower total bone marrow cellularity at day 70 compared to recipients receiving non-irradiated BM MNCs (Supplementary Figure S5A). Recipients of irradiated BM MNCs also had a higher percentage of circulating neutrophils (Supplementary Figure S5B) and higher BM ACE activity (Supplementary Figure S5C). In the analysis of the lung immune compartment, rats transplanted with irradiated BM MNCs had elevated total CD45^+^ and CD45^+^CD11b^+^ cells relative to recipients of non-irradiated BM MNCs (Figures 6B, C). Within the CD45^+^CD11b^+^ fraction, about 39% of cells were positive for ACE and no differences in ACE enrichment were observed between treatment groups (Figures 6D, E). However, the overall percentage of ACE^+^CD45^+^CD11b^+^ cells was elevated in the recipients of irradiated BM MNCs *versus* non-irradiated BM MNCs recipients (Figure 6E). Finally, we observed ACE activity within the lung CD45^+^ cell population to be significantly increased in the irradiated BM MNCs *versus* non-irradiated BM MNCs recipients (Figure 6F).

## Discussion

Biological sex-differences in normal tissue response to radiation injury have been reported by multiple labs (Jones et al., 2019; Fish et al., 2021; Sridharan et al., 2021; Brickey et al., 2022; Cosar et al., 2022; Orschell et al., 2022; Patterson et al., 2022; Andruska et al., 2023; Broustas et al., 2023; Gibbs et al., 2023; Singh et al., 2023) and the efficacy of candidate medical countermeasures (MCMs) for radiation toxicity have also been shown to vary based on biological sex (DeBo et al., 2015; Daniel et al., 2020; DiCarlo et al., 2021; Winters et al., 2023). Consistent with our prior studies, we have validated increased sensitivity to radiation-induced pneumonitis in male rats compared to age-matched female rats as evidenced by increased breathing rate, higher infiltration of immune cells in the lung, and reduced survival (Fish et al., 2021). Although male rats exhibited increased tryptase^+^ mast cells compared to female rats, we did not see a sex difference in collagen deposition by Masson’s trichrome staining at this time point during pneumonitis. Additional studies will be necessary to determine if there is a sex difference in the progression to late fibrotic lung injury.

We characterized expression of the RAS enzymes ACE and ACE2 to see if differential regulation of the RAS system may play a role in mediating this sex difference. The RAS system is known to be a crucial regulator of the pathogenesis of radiation-induced toxicities as ACE inhibitors have been shown to reduce multi-organ toxicity in animal models (Kma et al., 2012; Medhora et al., 2012; Fish et al., 2016; Mungunsukh et al., 2021) and the incidental use of lisinopril in cancer patients undergoing radiation therapy is associated with decreased normal tissue toxicity (Kharofa et al., 2012; Kerns et al., 2022). Additionally, we have recently demonstrated that pharmacologic agonism of ACE2 also significantly improves survival during pneumonitis (Gasperetti et al., 2022). In our prior studies, we demonstrated that either pharmacologic inhibition of ACE or pharmacologic activation of ACE2 regulate immune cell function (Gasperetti et al., 2022; Sharma et al., 2022). In the case of ACE inhibition with lisinopril, we observed a reduction of radiation-induced inflammatory response *in vivo* and a direct suppression of radiation-induced ROS generation in human CD14^+^ monocytes (Sharma et al., 2022). Whereas agonism of ACE2 was observed to promote hematopoietic reconstitution and increase survival during the acute radiation syndrome in female rats (Gasperetti et al., 2022). Together, these studies suggest the RAS plays a key role in mediating the immune response following radiation injury.

For these reasons, we evaluated the local expression of ACE and ACE2 in the lung following radiation injury. Here we observed both sex and radiation dependent changes in ACE and ACE2 expression. In the lung, ACE2 protein is increased in female rats relative to male rats both before and at 70 days following radiation, while ACE is significantly increased by radiation in male rats alone. The concurrent increase in ACE and lower basal ACE2 levels leads to an overall imbalance in the local RAS in the lungs of male rats. The radiation-induced changes in ACE and ACE2 expression are similar to findings in a hypertension rat model, where male rats were observed to have an imbalance in the relative levels of lung ACE and ACE2 levels pathways favoring activation of the proinflammatory ACE/AngII signaling pathway (Martins et al., 2021). Additionally, in adult mice ACE2 protein expression is significantly upregulated in the lungs of female *versus* male mice (Shahbaz et al., 2022). Importantly, we observed similar survival in males and females treated with ACE2 agonist DIZE which indicates pharmacologic correction of the radiation-induced ACE/ACE2 imbalance may abrogate sex-differences in radiosensitivity. Of note, ACE2 agonism with DIZE was capable of rescuing male rats from morbidity due to GI-ARS. Future studies will be necessary to evaluate the potential dysregulation of the gut RAS, but recent studies suggest intestinal ACE and ACE2 may play a regulatory role in the intestine (Ferreira-Duarte et al., 2023).

In the irradiated lung, we hypothesized the observed imbalance in ACE/ACE2 ratio was due in part to dysregulation of myeloid cell compartment as we previously observed lung infiltrating ACE-expressing myeloid cells are increased post-irradiation (Sharma et al., 2022). This observation led us to examine the bone marrow compartment following radiation injury in these same animals. Within the bone marrow at day 70, we observed a long-term suppression in total cellularity in both male and female rats. This is consistent with the long-term bone marrow dysfunction and chronic injury observed in prior rodent studies of long-term survivors of high dose irradiation (Chua et al., 2019; Gasperetti et al., 2023). However, in male rats alone, we observed a significant increase following radiation in the bone marrow myeloid compartment, higher numbers of ACE-expressing CD11b^+^ myeloid cells, and increased ACE activity. We report 5%-10% of CD11b myeloid cells express ACE which is line with prior characterization of the local bone marrow RAS in rats (Strawn et al., 2004). Interestingly, mouse models of bone marrow aging also show a similar pattern of increased myelopoiesis and upregulation of ACE protein (Chittimalli et al., 2023). As ACE expression in the myeloid compartment has previously been shown to regulate myelopoiesis, macrophage differentiation, and systemic immune function in both human and rodent models (Okwan-Duodu et al., 2010; Lin et al., 2011; Okwan-Duodu et al., 2019; Bueno et al., 2023), we surmised radiation-induced ACE activation in the bone marrow myeloid compartment may promote a systemic pro-inflammatory response that exacerbates radiation pneumonitis.

To investigate the potential connection between radiation injury to the bone marrow and systemic pro-inflammatory response, we then assessed whether lung immune infiltrate is increased in recipients of irradiated bone marrow compared to recipients of non-irradiated bone marrow. Relative to recipients of non-irradiated bone marrow, we observed irradiated recipients exhibit a greater increase in immune cell infiltration in the lung as well as higher numbers of ACE^+^ CD11b^+^ myeloid cells and increased ACE activity. One notable shortcoming of this study was that it was only performed with only female bone marrow donor and female recipient rats. We surmise the observed differences in immune cell infiltrate and ACE activity may have been more pronounced in male rats, but future studies will be necessary to confirm. Despite this shortcoming, this study confirms that long-term injury to the bone marrow alters the progression of radiation pneumonitis and increases ACE-expressing myeloid cell accumulation in the irradiated lung. These data this support the growing body of evidence that ACE-expressing myeloid cells regulate the pathogenesis of inflammatory diseases (Friedland et al., 1978; Danilov et al., 2003; Rutkowska-Zapala et al., 2015; Danilov et al., 2019; Cao et al., 2020; Tonon et al., 2022).

Finally, to our knowledge, this study is the first to report organ-specific sex-differences in the RAS following radiation injury. Sex differences in the RAS are well-established in other diseases and have been proposed to be a factor in the observed sex disparity in COVID-19 mortality (Gagliardi et al., 2020; Mukherjee and Pahan, 2021; Rocheleau et al., 2022; Xie et al., 2023). However, an important distinction that is not addressed by the current study is whether the observed increase in ACE^+^ myeloid cells in male rats is mainly due to increased myelopoiesis in male rats as monocyte differentiation in known to be increased in males and is thought to be driven by testosterone signaling through the androgen receptor (AR) in myeloid progenitor cells (Consiglio and Gollnick, 2020). Sex hormones are indeed likely to contribute to radiation sensitivity in this model as previous studies in the rat PBI model demonstrated no sex differences in radiation pneumonitis in pre-pubertal rats (Medhora et al., 2019). Furthermore, female sex hormones induce ACE2 activity (Reis and Reis, 2020) and prior studies have shown that activation of the ACE2/Ang1-7/MasR pathway via Ang1-7 administration decreases myelopoiesis and corrects for aging associated increases in bone marrow inflammation (Chittimalli et al., 2023).

Together, these data highlight the importance of sex as a biological variable in the development of medical countermeasures for treatment radiation injuries. Future studies will be necessary to determine if the response to therapeutics targeting the RAS pathway is sex dependent.

## Data Availability

The original contributions presented in the study are included in the article/Supplementary Material, further inquiries can be directed to the corresponding author.

## Supplementary material

Supplementary Figure S1 Masson’s trichrome stained lung sections. Left, representative photomicrographs of lung tissue from female and male rats at day 70 day in 0 Gy control and following 12.5 Gy PBI. Right, quantification of the collagen-positive fibrotic region. The error bars indicate the standard error of the mean *****p* < 0.0001.

Supplementary Figure S2 Whole lung transcriptional expression of *Ace* and *Ace2*. mRNA expression of *Ace* and *Ace2* mRNA in the whole lung tissue at day 42 post-irradiation. (N = 5 rats/group).

Supplementary Figure S3 Uncropped Western blots for ACE, ACE2, and GAPDH.

Supplementary Figure S4 ACE2 Enzymatic Activity in the lungs of DIZE treated rats. ACE2 enzymatic activity was measured in the whole lung of control and irradiated (Day 50) male rats treated with DIZE.

Supplementary Figure S5 Recipients of irradiated bone marrow exhibit elevated BM ACE activity. **(A)** BM cellularity 13 Gy TBI rats transplanted with either control or irradiated (7.75 Gy) BM MNCs on day 70. **(B)** Percent peripheral blood neutrophils (NEU) at day 70 in the recipient rats. **(C)** Enzymatic whole BM ACE activity at day 70. **p* < 0.05; ***p* < 0.01.

## References

[B1] Andruska N. Schlaak R. A. Frei A. Schottstaedt A. M. Lin C. Y. Fish B. L. (2023). Differences in radiation-induced heart dysfunction in male versus female rats. Int. J. Radiat. Biol. 2023, 1–13. 10.1080/09553002.2023.2194404 PMC10431914 36971580

[B2] Barhoumi T. Alghanem B. Shaibah H. Mansour F. A. Alamri H. S. Akiel M. A. (2021). SARS-CoV-2 coronavirus spike protein-induced apoptosis, inflammatory, and oxidative stress responses in THP-1-like-macrophages: Potential role of angiotensin-converting enzyme inhibitor (perindopril). Front. Immunol. 12, 728896. 10.3389/fimmu.2021.728896 34616396 PMC8488399

[B3] Brickey W. J. Thompson M. A. Sheng Z. Li Z. Owzar K. Ting J. P. Y. (2022). Re-examination of the exacerbating effect of inflammasome components during radiation injury. Radiat. Res. 197 (2), 199–204. 10.1667/RADE-21-00142.1 34855933 PMC8982344

[B4] Broustas C. G. Shuryak I. Duval A. J. Amundson S. A. (2023). Effect of age and sex on gene expression-based radiation biodosimetry using mouse peripheral blood. Cytogenet Genome Res. 2023, 530172. 10.1159/000530172 PMC10585707 36928338

[B5] Bueno V. Forones N. M. Pawelec G. (2023). Alternative chemotherapies: Angiotensin-converting enzyme inhibitors reduce myeloid-derived suppressor cells to benefit older patients with colorectal cancer. Front. Biosci. (Landmark Ed. 28 (1), 2. 10.31083/j.fbl2801002 36722279

[B6] Cao D. Y. Saito S. Veiras L. C. Okwan-Duodu D. Bernstein E. A. Giani J. F. (2020). Role of angiotensin-converting enzyme in myeloid cell immune responses. Cell Mol. Biol. Lett. 25, 31. 10.1186/s11658-020-00225-w 32508938 PMC7249647

[B7] Chittimalli K. Jahan J. Sakamuri A. Weyrick H. Winkle W. Adkins S. (2023). Reversal of aging-associated increase in myelopoiesis and expression of alarmins by angiotensin-(1-7). Sci. Rep. 13 (1), 2543. 10.1038/s41598-023-29853-w 36782016 PMC9925828

[B8] Chua H. L. Plett P. A. Fisher A. Sampson C. H. Vemula S. Feng H. (2019). Lifelong residual bone marrow damage in murine survivors of the hematopoietic acute radiation syndrome (H-ars): A compilation of studies comprising the Indiana university experience. Health Phys. 116 (4), 546–557. 10.1097/HP.0000000000000950 30789496 PMC6388630

[B9] Consiglio C. R. Gollnick S. O. (2020). Androgen receptor signaling positively regulates monocytic development. Front. Immunol. 11, 519383. 10.3389/fimmu.2020.519383 33193298 PMC7604537

[B10] Cosar R. Ozen A. Tastekin E. Sut N. Cakina S. Demir S. (2022). Does gender difference effect radiation-induced lung toxicity? An experimental study by genetic and histopathological predictors. Radiat. Res. 197 (3), 280–288. 10.1667/RADE-21-00075.1 34735567

[B11] Daniel A. R. Lee C. L. Oh P. Luo L. Ma Y. Kirsch D. G. (2020). Inhibiting glycogen synthase kinase-3 mitigates the hematopoietic acute radiation syndrome in a sex- and strain-dependent manner in mice. Health Phys. 119 (3), 315–321. 10.1097/HP.0000000000001243 32175929 PMC7398824

[B12] Danilov S. M. Metzger R. Klieser E. Sotlar K. Trakht I. N. Garcia J. G. N. (2019). Tissue ACE phenotyping in lung cancer. PLoS One 14 (12), e0226553. 10.1371/journal.pone.0226553 31877149 PMC6932779

[B13] Danilov S. M. Sadovnikova E. Scharenborg N. Balyasnikova I. V. Svinareva D. A. Semikina E. L. (2003). Angiotensin-converting enzyme (CD143) is abundantly expressed by dendritic cells and discriminates human monocyte-derived dendritic cells from acute myeloid leukemia-derived dendritic cells. Exp. Hematol. 31 (12), 1301–1309. 10.1016/j.exphem.2003.08.018 14662338

[B14] DeBo R. J. Register T. C. Caudell D. L. Sempowski G. D. Dugan G. Gray S. (2015). Molecular and cellular profiling of acute responses to total body radiation exposure in ovariectomized female cynomolgus macaques. Int. J. Radiat. Biol. 91 (6), 510–518. 10.3109/09553002.2015.1028597 25786585 PMC4566160

[B15] DiCarlo A. L. Perez Horta Z. Rios C. I. Satyamitra M. M. Taliaferro L. P. Cassatt D. R. (2021). Study logistics that can impact medical countermeasure efficacy testing in mouse models of radiation injury. Int. J. Radiat. Biol. 97 (1), S151–S167. 10.1080/09553002.2020.1820599 32909878 PMC7987915

[B16] Ferreira-Duarte M. Oliveira L. C. G. Quintas C. Esteves-Monteiro M. Duarte-Araujo M. Sousa T. (2023). ACE and ACE2 catalytic activity in the fecal content along the gut. Neurogastroenterol. Motil. 2023, e14598. 10.1111/nmo.14598 37052403

[B17] Fish B. L. Gao F. Narayanan J. Bergom C. Jacobs E. R. Cohen E. P. (2016). Combined hydration and antibiotics with lisinopril to mitigate acute and delayed high-dose radiation injuries to multiple organs. Health Phys. 111 (5), 410–419. 10.1097/HP.0000000000000554 27682899 PMC5065284

[B18] Fish B. L. MacVittie T. J. Gao F. Narayanan J. Gasperetti T. Scholler D. (2021). Rat models of partial-body irradiation with bone marrow-sparing (Leg-out PBI) designed for FDA approval of countermeasures for mitigation of acute and delayed injuries by radiation. Health Phys. 121 (4), 419–433. 10.1097/HP.0000000000001444 34546222 PMC8577554

[B19] Friedland J. Setton C. Silverstein E. (1978). Induction of angiotensin converting enzyme in human monocytes in culture. Biochem. Biophys. Res. Commun. 83 (3), 843–849. 10.1016/0006-291x(78)91471-7 213075

[B20] Gagliardi M. C. Tieri P. Ortona E. Ruggieri A. (2020). ACE2 expression and sex disparity in COVID-19. Cell Death Discov. 6, 37. 10.1038/s41420-020-0276-1 32499922 PMC7248455

[B21] Gasperetti T. Frei A. Prasad Sharma G. Pierce L. Veley D. Szalewski N. (2023). Delayed renal injury in survivors of hematologic acute radiation syndrome. Int. J. Radiat. Biol. 2023, 1–9. 10.1080/09553002.2023.2170491 PMC10313734 36688956

[B22] Gasperetti T. Miller T. Gao F. Narayanan J. Jacobs E. R. Szabo A. (2021). Corrigendum: Polypharmacy to mitigate acute and delayed radiation syndromes. Front. Pharmacol. 12 (1027), 741485. 10.3389/fphar.2021.741485 34512365 PMC8424069

[B23] Gasperetti T. Sharma G. P. Frei A. C. Pierce L. Veley D. Szalewski N. (2022). Mitigation of multi-organ radiation injury with ACE2 agonist diminazene aceturate. Radiat. Res. 198 (4), 325–335. 10.1667/RADE-22-00055.1 35904437 PMC9641750

[B24] Geng F. Chen J. Tang S. Azzam E. I. Zhang J. Zhang S. (2022). Additional evidence for commonalities between COVID-19 and radiation injury: Novel insight into COVID-19 candidate drugs. Radiat. Res. 198 (3), 306–317. 10.1667/RADE-22-00058.1 35834824

[B25] Gibbs A. Gupta P. Mali B. Poirier Y. Gopalakrishnan M. Newman D. (2023). A C57L/J mouse model of the delayed effects of acute radiation exposure in the context of evolving multi-organ dysfunction and failure after total-body irradiation with 2.5% bone marrow sparing. Radiat. Res. 199 (4), 319–335. 10.1667/RADE-22-00178.1 36857032 PMC10289057

[B26] Haga S. Yamamoto N. Nakai-Murakami C. Osawa Y. Tokunaga K. Sata T. (2008). Modulation of TNF-alpha-converting enzyme by the spike protein of SARS-CoV and ACE2 induces TNF-alpha production and facilitates viral entry. Proc. Natl. Acad. Sci. U. S. A. 105 (22), 7809–7814. 10.1073/pnas.0711241105 18490652 PMC2409424

[B27] Imai Y. Kuba K. Rao S. Huan Y. Guo F. Guan B. (2005). Angiotensin-converting enzyme 2 protects from severe acute lung failure. Nature 436 (7047), 112–116. 10.1038/nature03712 16001071 PMC7094998

[B28] Jones J. W. Alloush J. Sellamuthu R. Chua H. L. MacVittie T. J. Orschell C. M. (2019). Effect of sex on biomarker response in a mouse model of the hematopoietic acute radiation syndrome. Health Phys. 116 (4), 484–502. 10.1097/HP.0000000000000961 30681425 PMC6384137

[B29] Kerns S. L. Amidon Morlang A. Lee S. M. Peterson D. R. Marples B. Zhang H. (2022). Use of angiotensin converting enzyme inhibitors is associated with reduced risk of late bladder toxicity following radiotherapy for prostate cancer. Radiother. Oncol. 168, 75–82. 10.1016/j.radonc.2022.01.014 35077710 PMC8986577

[B30] Kharofa J. Cohen E. P. Tomic R. Xiang Q. Gore E. (2012). Decreased risk of radiation pneumonitis with incidental concurrent use of angiotensin-converting enzyme inhibitors and thoracic radiation therapy. Int. J. Radiat. Oncol. Biol. Phys. 84 (1), 238–243. 10.1016/j.ijrobp.2011.11.013 22300564

[B31] Kim J. H. Brown S. L. Kolozsvary A. Jenrow K. A. Ryu S. Rosenblum M. L. (2004). Modification of radiation injury by ramipril, inhibitor of angiotensin-converting enzyme, on optic neuropathy in the rat. Radiat. Res. 161 (2), 137–142. 10.1667/rr3124 14731077

[B32] Kma L. Gao F. Fish B. L. Moulder J. E. Jacobs E. R. Medhora M. (2012). Angiotensin converting enzyme inhibitors mitigate collagen synthesis induced by a single dose of radiation to the whole thorax. J. Radiat. Res. 53 (1), 10–17. 10.1269/jrr.11035 22302041 PMC3616750

[B33] Krystel-Whittemore M. Dileepan K. N. WoodMast Cell J. G. (2015). Mast cell: A multi-functional master cell. Front. Immunol. 6, 620. 10.3389/fimmu.2015.00620 26779180 PMC4701915

[B34] Lin C. Datta V. Okwan-Duodu D. Chen X. Fuchs S. Alsabeh R. (2011). Angiotensin-converting enzyme is required for normal myelopoiesis. FASEB J. 25 (4), 1145–1155. 10.1096/fj.10-169433 21148418 PMC3058713

[B35] Martins F. L. Tavares C. A. M. Malagrino P. A. Rentz T. Benetti A. Rios T. M. S. (2021). Sex differences in the lung ACE/ACE2 balance in hypertensive rats. Biosci. Rep. 41 (12). 10.1042/BSR20211201 PMC8655502 34751382

[B36] McCart E. A. Lee Y. H. Jha J. Mungunsukh O. Rittase W. B. Summers T. A. (2019). Delayed captopril administration mitigates hematopoietic injury in a murine model of total body irradiation. Sci. Rep. 9 (1), 2198. 10.1038/s41598-019-38651-2 30778109 PMC6379397

[B37] Medhora M. Gao F. Gasperetti T. Narayanan J. Khan A. H. Jacobs E. R. (2019). Delayed effects of acute radiation exposure (deare) in juvenile and old rats: Mitigation by lisinopril. Health Phys. 116 (4), 529–545. 10.1097/HP.0000000000000920 30624354 PMC6384142

[B38] Medhora M. Gao F. Jacobs E. R. Moulder J. E. (2012). Radiation damage to the lung: Mitigation by angiotensin-converting enzyme (ACE) inhibitors. Respirology 17 (1), 66–71. 10.1111/j.1440-1843.2011.02092.x 22023053 PMC3245332

[B39] Miličić Stanić B. Maddox S. de Souza A. M. Wu X. Mehranfard D. Ji H. (2021). Male bias in ACE2 basic science research: Missed opportunity for discovery in the time of COVID-19. Am. J. Physiology-Regulatory, Integr. Comp. Physiology 320 (6), R925–R937. 10.1152/ajpregu.00356.2020 PMC8203415 33848207

[B40] Moulder J. E. Fish B. L. Cohen E. P. (2007). Treatment of radiation nephropathy with ACE inhibitors and AII type-1 and type-2 receptor antagonists. Curr. Pharm. Des. 13 (13), 1317–1325. 10.2174/138161207780618821 17506717

[B41] Mukherjee S. Pahan K. (2021). Is COVID-19 gender-sensitive? J. Neuroimmune Pharmacol. 16 (1), 38–47. 10.1007/s11481-020-09974-z 33405098 PMC7786186

[B42] Mungunsukh O. George J. McCart E. A. Snow A. L. Mattapallil J. J. Mog S. R. (2021). Captopril reduces lung inflammation and accelerated senescence in response to thoracic radiation in mice. J. Radiat. Res. 62 (2), 236–248. 10.1093/jrr/rraa142 33616187 PMC7948861

[B43] Okwan-Duodu D. Datta V. Shen X. Z. Goodridge H. S. Bernstein E. A. Fuchs S. (2010). Angiotensin-converting enzyme overexpression in mouse myelomonocytic cells augments resistance to Listeria and methicillin-resistant *Staphylococcus aureus* . J. Biol. Chem. 285 (50), 39051–39060. 10.1074/jbc.M110.163782 20937811 PMC2998083

[B44] Okwan-Duodu D. Weiss D. Peng Z. Veiras L. C. Cao D. Y. Saito S. (2019). Overexpression of myeloid angiotensin-converting enzyme (ACE) reduces atherosclerosis. Biochem. Biophys. Res. Commun. 520 (3), 573–579. 10.1016/j.bbrc.2019.10.078 31615657 PMC6916669

[B45] Orschell C. M. Wu T. Patterson A. M. (2022). Impact of age, sex, and genetic diversity in murine models of the hematopoietic acute radiation syndrome (H-ars) and the delayed effects of acute radiation exposure (DEARE). Curr. Stem Cell Rep. 8 (3), 139–149. 10.1007/s40778-022-00214-z 36798890 PMC9928166

[B46] Patterson A. M. Vemula S. Plett P. A. Sampson C. H. Chua H. L. Fisher A. (2022). Age and sex divergence in hematopoietic radiosensitivity in aged mouse models of the hematopoietic acute radiation syndrome. Radiat. Res. 198 (3), 221–242. 10.1667/RADE-22-00071.1 35834823 PMC9512046

[B47] Qi Y. Zhang J. Cole-Jeffrey C. T. Shenoy V. Espejo A. Hanna M. (2013). Diminazene aceturate enhances angiotensin-converting enzyme 2 activity and attenuates ischemia-induced cardiac pathophysiology. Hypertension 62 (4), 746–752. 10.1161/HYPERTENSIONAHA.113.01337 23959549 PMC3881360

[B48] Reis F. M. Reis A. M. (2020). Angiotensin-converting enzyme 2 (ACE2), angiotensin-(1-7) and Mas receptor in gonadal and reproductive functions. Clin. Sci. (Lond). 134 (22), 2929–2941. 10.1042/CS20200865 33196086

[B49] Rios C. I. Cassatt D. R. Hollingsworth B. A. Satyamitra M. M. Tadesse Y. S. Taliaferro L. P. (2021). Commonalities between COVID-19 and radiation injury. Radiat. Res. 195 (1), 1–24. 10.1667/RADE-20-00188.1 33064832 PMC7861125

[B50] Rocheleau G. L. Y. Lee T. Mohammed Y. Goodlett D. Burns K. Cheng M. P. (2022). Renin-angiotensin system pathway therapeutics associated with improved outcomes in males hospitalized with COVID-19. Crit. Care Med. 50 (9), 1306–1317. 10.1097/CCM.0000000000005589 35607951 PMC9380153

[B51] Rutkowska-Zapala M. Suski M. Szatanek R. Lenart M. Weglarczyk K. Olszanecki R. (2015). Human monocyte subsets exhibit divergent angiotensin I-converting activity. Clin. Exp. Immunol. 181 (1), 126–132. 10.1111/cei.12612 25707554 PMC4469162

[B52] Ryu S. Kolozsvary A. Jenrow K. A. Brown S. L. Kim J. H. (2007). Mitigation of radiation-induced optic neuropathy in rats by ACE inhibitor ramipril: Importance of ramipril dose and treatment time. J. Neurooncol 82 (2), 119–124. 10.1007/s11060-006-9256-4 17004100

[B53] Schramm A. Matusik P. Osmenda G. Guzik T. J. (2012). Targeting NADPH oxidases in vascular pharmacology. Vasc. Pharmacol. 56 (5-6), 216–231. 10.1016/j.vph.2012.02.012 PMC3378316 22405985

[B54] Shahbaz S. Oyegbami O. Saito S. Osman M. Sligl W. Elahi S. (2022). Differential effects of age, sex and dexamethasone therapy on ACE2/TMPRSS2 expression and susceptibility to SARS-CoV-2 infection. Front. Immunol. 13, 1021928. 10.3389/fimmu.2022.1021928 36405732 PMC9671168

[B55] Sharma G. P. Fish B. L. Frei A. C. Narayanan J. Gasperetti T. Scholler D. (2022). Pharmacologic ACE-inhibition mitigates radiation-induced pneumonitis by suppressing ACE-expressing lung myeloid cells. Int. J. Radiat. Oncol. Biol. Phys. 113, 177–191. 10.1016/j.ijrobp.2022.01.023 35093482 PMC9018504

[B56] Sharma G. P. Gurung S. K. Inam A. Nigam L. Bist A. Mohapatra D. (2019). CID-6033590 inhibits p38MAPK pathway and induces S-phase cell cycle arrest and apoptosis in DU145 and PC-3 cells. Toxicol. Vitro 60, 420–436. 10.1016/j.tiv.2019.06.003 31175925

[B57] Singh V. K. Carpenter A. D. Janocha B. L. Petrus S. A. Fatanmi O. O. Wise S. Y. (2023). Radiosensitivity of rhesus nonhuman primates: Consideration of sex, supportive care, body weight and age at time of exposure. Expert Opin. Drug Discov. 2023, 2205123. 10.1080/17460441.2023.2205123 PMC10330264 37073409

[B58] Sridharan V. Johnson K. A. Landes R. D. Cao M. Singh P. Wagoner G. (2021). Sex-dependent effects of genetic upregulation of activated protein C on delayed effects of acute radiation exposure in the mouse heart, small intestine, and skin. PLoS One 16 (5), e0252142. 10.1371/journal.pone.0252142 34029348 PMC8143413

[B59] Sriram K. Insel P. A. (2020). A hypothesis for pathobiology and treatment of COVID-19: The centrality of ACE1/ACE2 imbalance. Br. J. Pharmacol. 177 (21), 4825–4844. 10.1111/bph.15082 32333398 PMC7572451

[B60] Strawn W. B. Richmond R. S. Ann Tallant E. Gallagher P. E. Ferrario C. M. (2004). Renin-angiotensin system expression in rat bone marrow haematopoietic and stromal cells. Br. J. Haematol. 126 (1), 120–126. 10.1111/j.1365-2141.2004.04998.x 15198742

[B61] Szabo S. Ghosh S. N. Fish B. L. Bodiga S. Tomic R. Kumar G. (2010). Cellular inflammatory infiltrate in pneumonitis induced by a single moderate dose of thoracic x radiation in rats. Radiat. Res. 173 (4), 545–556. 10.1667/RR1753.1 20334527 PMC2904484

[B62] Tonon F. Candido R. Toffoli B. Tommasi E. Cortello T. Fabris B. (2022). Type 1 diabetes is associated with significant changes of ACE and ACE2 expression in peripheral blood mononuclear cells. Nutr. Metab. Cardiovasc Dis. 32 (5), 1275–1282. 10.1016/j.numecd.2022.01.029 35260304

[B63] van der Veen S. J. Ghobadi G. de Boer R. A. Faber H. Cannon M. V. Nagle P. W. (2015). ACE inhibition attenuates radiation-induced cardiopulmonary damage. Radiother. Oncol. 114 (1), 96–103. 10.1016/j.radonc.2014.11.017 25465731

[B64] Ward H. E. Kemsley L. Davies L. Holecek M. Berend N. (1993). The pulmonary response to sublethal thoracic irradiation in the rat. Radiat. Res. 136 (1), 15–21. 10.2307/3578634 8210333

[B65] Ward W. F. Molteni A. Hinz J. M. (1990). Captopril reduces collagen and mast cell accumulation in irradiated rat lung. Int. J. Radiat. Oncology Biology Phys. 19 (6), 1405–1409. 10.1016/0360-3016(90)90351-j 2262365

[B66] Watanabe S. Watanabe K. Oishi T. Aiba M. Kageyama K. (1974). Mast cells in the rat alveolar septa undergoing fibrosis after ionizing irradiation. Ultrastructural and histochemical studies. Lab. Invest. 31 (5), 555–567. 4139390

[B67] Winters T. A. Cassatt D. R. Harrison-Peters J. R. Hollingsworth B. A. Rios C. I. Satyamitra M. M. (2023). Considerations of medical preparedness to assess and treat various populations during a radiation public health emergency. Radiat. Res. 199 (3), 301–318. 10.1667/RADE-22-00148.1 36656560 PMC10120400

[B68] Xie J. Huang Q. F. Zhang Z. Dong Y. Xu H. Cao Y. (2023). Angiotensin-converting enzyme 2 in human plasma and lung tissue. Blood Press 32 (1), 6–15. 10.1080/08037051.2022.2154745 36495008

