# Supplementary figures and images for: Biological sex differences in renin angiotensin system enzymes ACE and ACE2 regulate normal tissue response to radiation injury
Source: Front Physiol. 2023 May 19;14:1191237. doi: 10.3389/fphys.2023.1191237 (PMC10235526; doi:10.3389/fphys.2023.1191237)

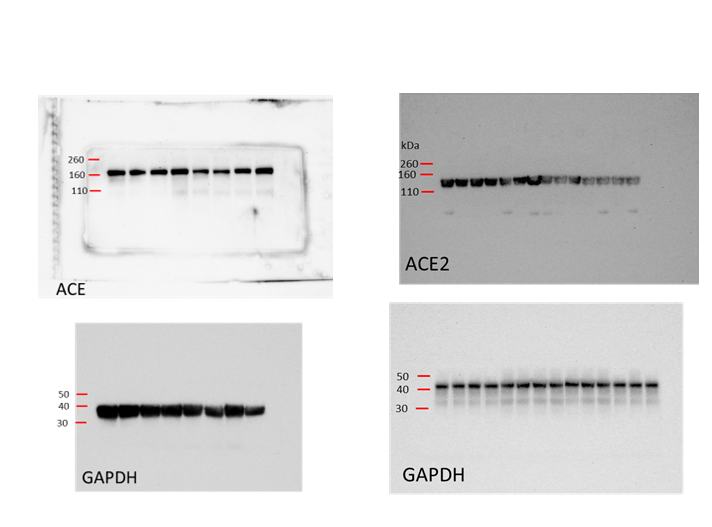

Supplement: Supplementary file 1 [file Image3.TIF]

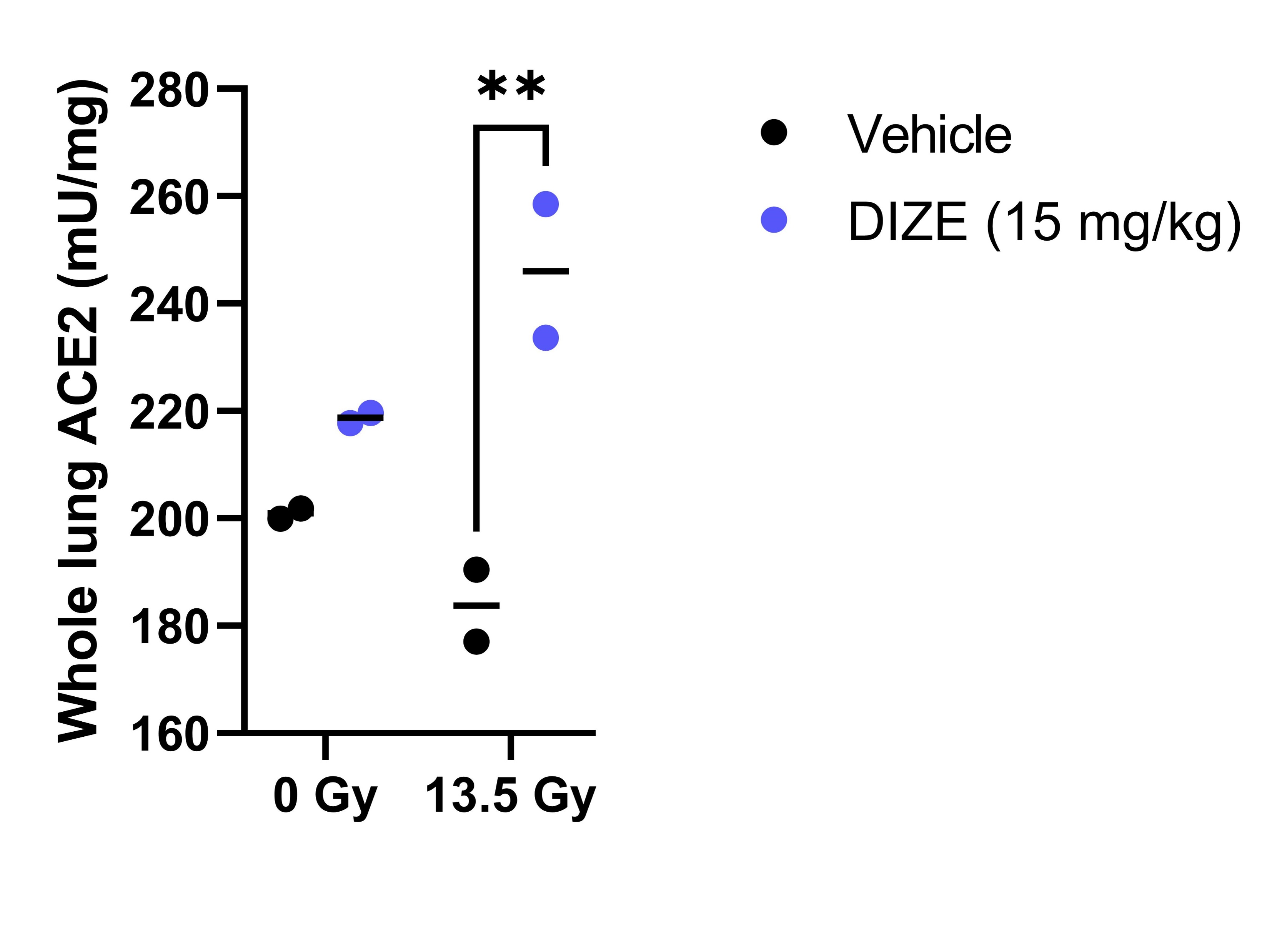

Supplement: Supplementary file 2 [file Image4.TIF]

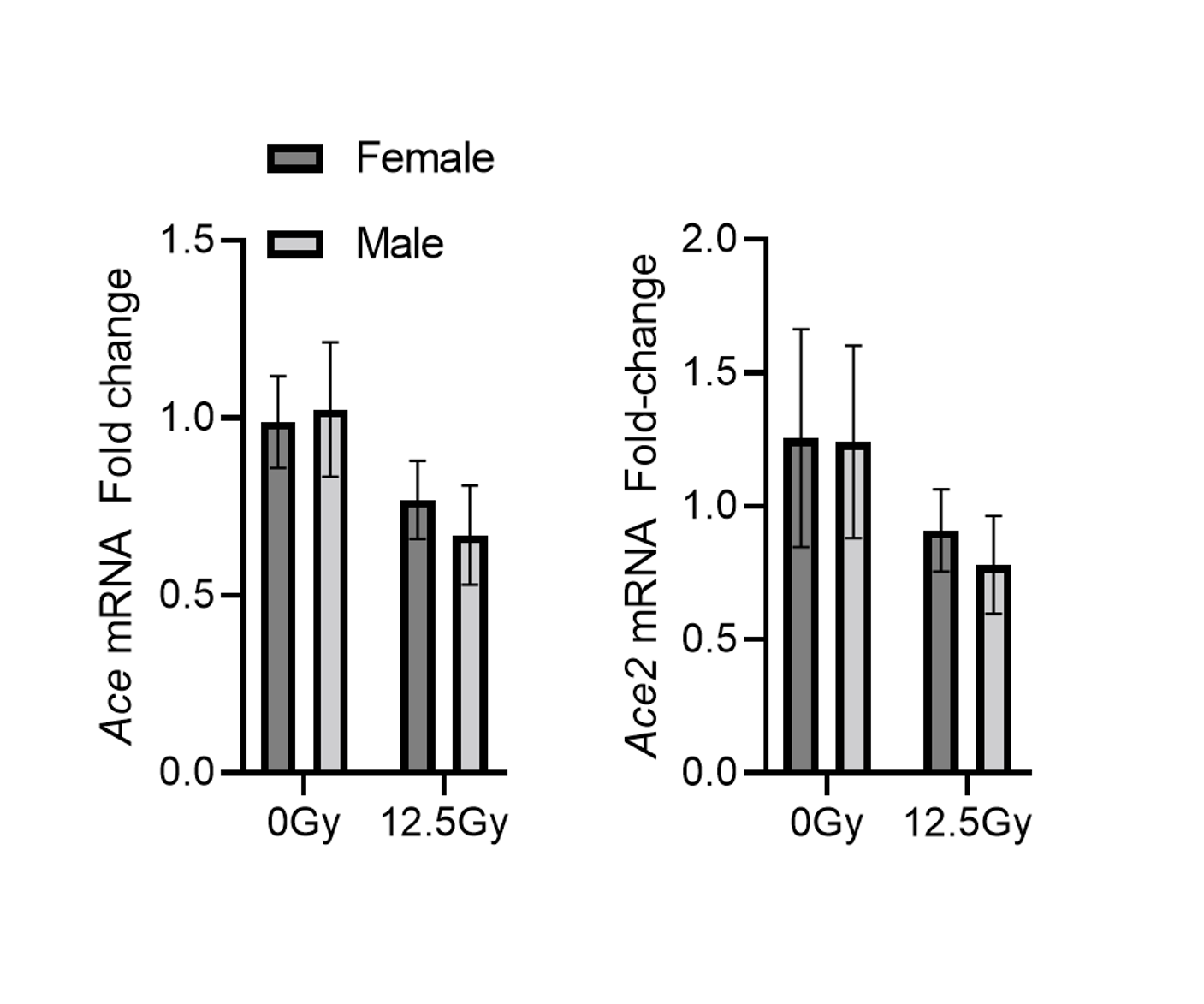

Supplement: Supplementary file 3 [file Image2.TIF]

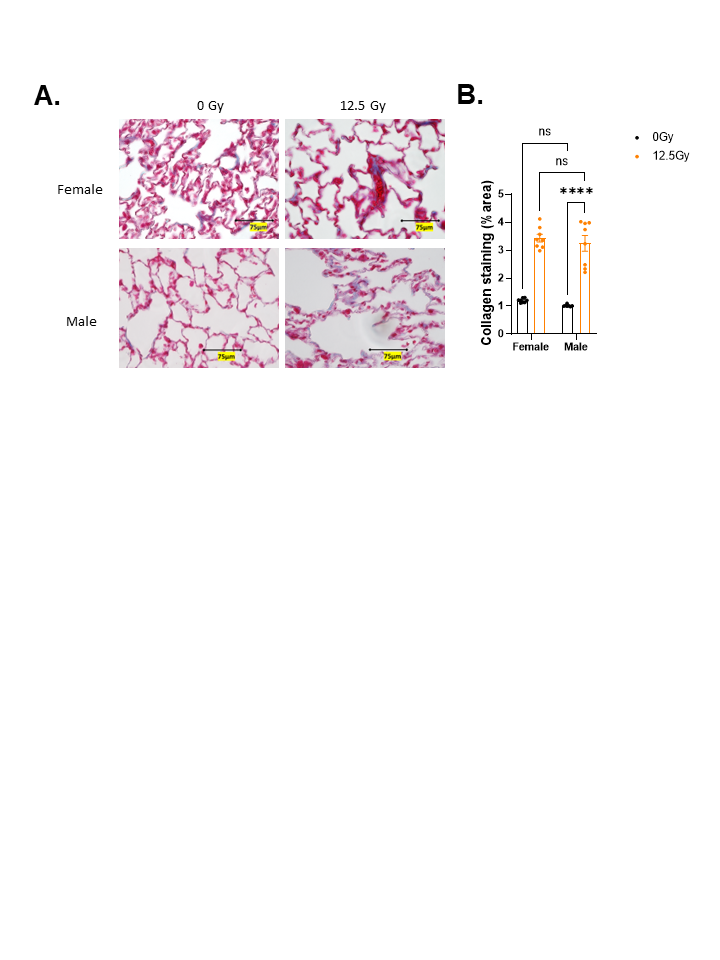

Supplement: Supplementary file 4 [file Image1.TIF]

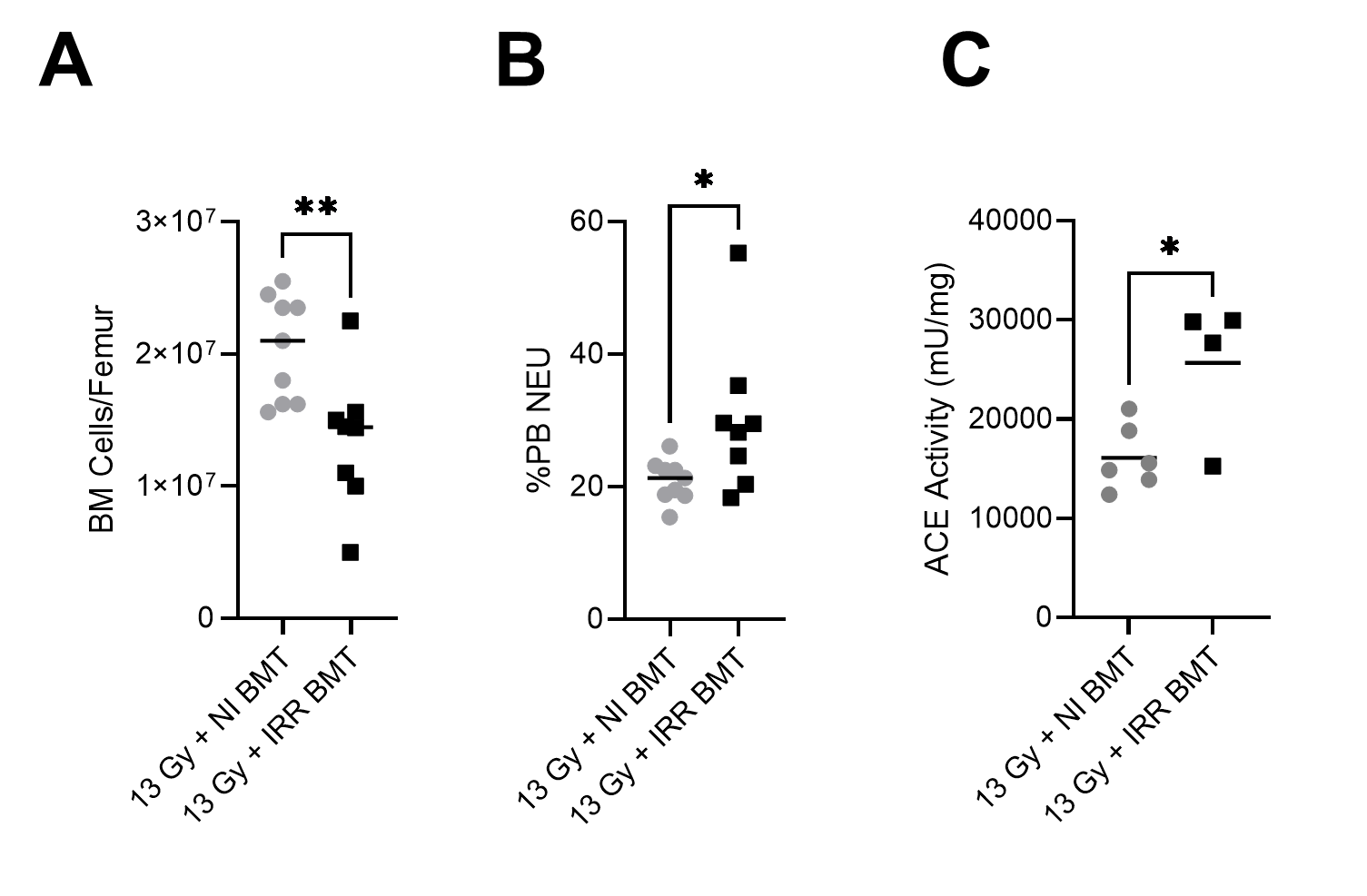

Supplement: Supplementary file 5 [file Image5.TIF]
